# Current status of innovative behaviors among nurses in public hospitals and its influencing factors: a cross-sectional study

**DOI:** 10.3389/fpsyg.2026.1742276

**Published:** 2026-03-03

**Authors:** Yimeng Zhu, Huihui Lin, Jielan Zhong, Xiaodi Li, Xiangqi Fu, Jiani Du, Yuhe Liu, Sheng Wang

**Affiliations:** 1 School of Public Health and Nursing, Hangzhou Normal University, Hangzhou, China; 2 Department of Cardiology, The Second Affiliated Hospital of Wenzhou Medical University, Wenzhou, China

**Keywords:** creative self-efficacy, innovative behavior, latent profile, nurses, paradoxical leadership, public hospitals, random forest, work engagement

## Abstract

**Objective:**

Nursing innovation significantly enhances the quality, efficiency, and effectiveness of nursing services, enabling nurses to cultivate a sense of occupational accomplishment and strengthen their professional competence. Previous studies only examined the factors of nurses’ innovative behaviors, and these factors were not ranked according to their importance. The purpose of this study is to identify the most important influencing factors that different categories of nurses’ innovative behaviors using comprehensive measures based on the conservation of resources theory.

**Methods:**

From August to December 2024, the survey was conducted among 832 nurses from 6 public tertiary hospitals in China. The data were analysed using Latent Profile Analysis, Pearson’s *χ*^2^ test, ANOVA and the random forest algorithm.

**Results:**

Overall, this study identified three subgroups of nurses’ innovative behavior: Innovation-Conservative, Innovation-Exploratory, and Innovation-Driven. Moreover, adequate creative self-efficacy, work engagement, and paradoxical leadership are the most important factors influencing all subgroups of nurse innovative behavior. However, the importance of secondary factors varied; for example, working years and average monthly income were critical for the Innovation-Conservative, while scheduling satisfaction was a key factor for Innovation-Exploratory. Additionally, innovative experience ranks relatively low.

**Conclusion:**

The innovative behaviors of nurses are slightly above the average of previous studies. Nursing managers should first enhance their paradoxical leadership to improve nurses’ creative self-efficacy and work engagement. Then, targeted intervention measures are implemented based on the characteristics of each group to enhance the professional quality and economic security of Innovation-Conservative and provide flexible and humanized scheduling for the Innovation-Exploratory, thereby effectively promoting the innovation of the entire nursing team.

## 1 Introduction

Innovation is considered a vital resource or ability, with nurses expected to enhance innovative behaviors to ensure organizational sustainability (Dess and Picken, 2000; Newman et al., 2020). Nurses are leading and developing solutions to complex problems requiring future-focused thinking, creativity, and expansive innovative frameworks to advance health (Beaudet, 2024). Currently, the wide application of digital technology such as artificial intelligence, mobile health platforms and big data has created new possibilities for the transformation of the nursing field. In 2025, the International Council of Nurses (ICN) redefined the concept of nurse, explicitly positioning innovation as a responsibility to address global health challenges through evidence-based technological and systemic advancements (International Council of Nurses, 2025). Innovation in the field of nursing can effectively improve healthcare efficiency, reduce medical errors, optimize resource allocation, and refine clinical workflows. For example, innovative nursing processes ensure accurate care implementation, decrease adverse patient events, shorten hospital stays, and reduce costs. Successful innovation implementation also affirms nursing professionals’ value, bolsters confidence, and promotes the advancement of the nursing discipline to a higher level (Murdock, 2025). Consequently, nursing innovation holds paramount importance. However, despite this recognition, nurses face significant barriers, including severe staffing shortages, excessive workloads, and time poverty that stifle their capacity for proactive, extra-role innovative behaviors, leading to a paradox where the demand for innovation coexists with structural constraints that suppress it. Research on individual innovative behavior remains scarce in nursing compared to information technology and knowledge-intensive industries. Studies indicate nurses’ innovative behavior typically operates at moderate levels, suggesting significant potential for improvement (Wang et al., 2024; Wang et al., 2024).

This study is grounded in the Conservation of Resources (COR) theory, proposed by Hobfoll (1989). It has now been widely applied in research fields such as management, medicine, psychology, and occupational health to explore the mechanism of resources in an individual’s response to stress stimulation. Furthermore, it is the desire to defend, conserve, and acquire these valued resources which motivates human behavior in the face of stress (Holmgreen et al., 2017). These resources are classified into four categories: object resources (e.g., living environment and work), conditional resources (e.g., social relationships, qualifications and experience), personal resources (e.g., self-efficacy and self-esteem), and energy resources (e.g., time, money, knowledge and social support). Psychological stress occurs when resources are threatened with loss or have actually been lost. On the contrary, when nurses have sufficient personal and conditional resources (e.g., high creative self-efficacy and supportive leadership) and the loss of resources can be borne, they will be more likely to participate in innovation, which requires the investment of resources such as time, energy and effort. Therefore, the COR theory provides a perspective for understanding the mechanisms by which various factors influence nurses’ innovative behaviors.

The influencing factors of nurses’ innovative behaviors are complex and diverse, and can be roughly divided into organizational factors and individual factors. Organizational factors include organizational climate (Lv et al., 2021), working environment (Akbari et al., 2020) and leadership style (Jønsson et al., 2021), while personal factors include demographic and sociological characteristics, personality traits and psychological factors. Paradoxical leadership is an important organizational resource that involves seemingly contradictory yet interrelated behaviors to simultaneously meet competing demands. Some studies have found that managers with paradoxical leadership can stimulate the potential of nurses’ innovative behaviors (Qing et al., 2023). In the field of nursing, nurses need to follow standardized nursing procedures while also providing personalized care based on the different characteristics of patients. While pursuing efficient medical services, it is also necessary to ensure the safety and quality of nursing. Nursing managers with this leadership style can effectively balance tensions, grant autonomy while maintaining control, or comply with the regulations while allowing flexibility, creating an atmosphere of psychological support. Secondly, creative self-efficacy, as a critical personal resource, also profoundly influences the innovation capabilities of nurses, which is derived from Bandura’s more general concept of self-efficacy (Bandura et al., 1997), explained as a person’s prospective belief that they can organize and execute the necessary actions for a successful creative process, leading to a successful creative product within a specific context (Anderson et al., 2022). From the perspective of COR, a strong sense of self-efficacy in innovation represents abundant personal resources, enabling individuals to be more resilient in the face of setbacks and more willing to invest their resources in challenging innovative tasks, as they are confident that their investments will yield positive returns. Besides, work engagement is a positive, fulfilling, and work-related mindset, characterized by vigor, dedication, and concentration (Zammitti et al., 2022; Hakanen and Kaltiainen, 2026). It represents a state of being energetic and full of motivation. Engaged nurses possess ample energy resources. As a particularly important variable in organizational research, work engagement positively predicts task performance, employee well-being outcomes, over and above alternative attitudes such as job satisfaction, organizational commitment (Arıcıoğlu and Timuroğlu, 2025; Mohammed et al., 2025), and organizational citizenship behavior (Loan and Nguyen, 2025; McManus et al., 2025). The COR theory holds that individuals with more resource reserves have a stronger ability to invest resources. Therefore, high work engagement enables nurses to have the energy to participate in extra role behaviors such as innovation in addition to fulfilling their own duties. Previous research has suggested that paradoxical leadership fosters heightened creative self-efficacy among employees, cultivating persistent positive self-perceptions (Wei et al., 2023). In such cases, both creative self-efficacy and work engagement are enhanced (Jung et al., 2022; Shehata et al., 2023). Creative self-efficacy increases employees’ willingness to actively participate in organization activities, which is a phenomenon that often occurs when employees are aware of positive feedback and fairness within the organization (Chughtai and Khalid, 2023). Al-Hamdan and Bani (2022) found that organizational support directly strengthens nurse work engagement. To a certain extent, organizational support can make a certain positive prediction of work engagement. Therefore, this study suggests that nurses’ innovative behaviors are influenced by paradoxical leadership, creative self-efficacy, and work engagement.

To explore more comprehensively the factors that may be related to nurses’ innovative behaviors, in addition to paradoxical leadership, creative self-efficacy and work engagement, some other variables were included in this study based on the COR theory, previous research, and clinical practice. First, demographic and occupational variables (working years, professional title, age, education level, marital status, average monthly income) were incorporated for their established links to innovative tendencies. Working years reflect accumulated clinical and interpersonal resources that support practice-based innovation (Duan et al., 2026). Professional title signifies occupational authority and resource access to implement extra role innovative behaviors (Fu et al., 2025). Age correlates with career stage and risk perception, shaping proactive engagement in new practices (Liu et al., 2024). Education level enhances cognitive resources for critical thinking and evidence-based innovation (Chen et al., 2020; Wang et al., 2025). Marital status is associate with social support networks, affecting resource allocation to beneficial behaviors that exceed job responsibilities (Nisar et al., 2024). Average monthly income is likely to reduce survival anxiety and boosts risk tolerance, key for resource investment in innovation. Second, satisfaction with scheduling was included as a conditional resource, which could help fostering work-life balance and perceived autonomy, supporting proactive innovative attempts by conserving physical and psychological resources (Stimpfel et al., 2025). Third, innovation experience variables (participation in scientific research training, experience of innovation, participation in academic conferences, publication of academic articles, participation in teaching innovation competitions) reflect past practical experience and capability development in academic and creative practice. These variables are closely associated with the formation of proactive work tendencies and the willingness to perform extra role behaviors such as innovation (Rylee and Cvanagh, 2023). Research training builds cognitive skills for generating innovative ideas (Huang et al., 2022). Academic conferences tend to provide external knowledge and peer support to stimulate innovation (Sun et al., 2024). Academic publication represents a formal output of innovative research that brings organizational recognition and professional reputation, which are valuable conditional resources (Henshall and Lewin, 2022). And teaching innovation competitions widely carried out in hospitals accumulate operational skills for translating ideas into practice.

Adopting Latent Profile Analysis (LPA) to identify subgroups of nurses based on their innovative behavior, and then employing Random Forest, a machine learning algorithm, to pinpoint the most critical factors predicting innovative behaviors in each subgroup. Latent profile analysis is a statistical analysis method that classifies different latent subgroups based on the response patterns of individuals on explicit variables, observes their population characteristics and heterogeneity. This individual-centered approach combined with machine learning, can provide a more detailed understanding of the heterogeneity among nurses’ innovative behaviors. It not only helps to offer targeted support to nurses in different subgroups and enhance their professional value, but also further optimizes the clinical outcomes and health prognosis of patients, providing a scientific basis for nursing management and training.

## 2 Materials and methods

### 2.1 Design

The cross-sectional design and convenience sampling methods were adopted, with the aim of evaluating the different subtypes of nurses’ innovative behavior by ranking the importance of determinants. We adhered to Strengthening the Reporting of Observational Studies in Epidemiology (STROBE) guidelines and methodology in reports of cross-sectional studies (details in Supplementary material).

### 2.2 Participants

In the context of this cross-sectional investigation, data were collected from August through December 2024, utilizing the professional survey platform, Questionnaire Star. The participants were recruited from six tertiary public hospitals in Zhejiang province, China.

The criteria for participant inclusion were stringently defined. Inclusion criteria were as follows: (1) holding professional qualification certificates for nurses issued by the People’s Republic of China; (2) working with the head nurse of the department for 1 year or more; (3) engaging in front-line clinical nursing work; (4) participating in this study voluntarily. The exclusion criteria were as follows: (1) nurses who were on vacation at the time of the survey, out for training, sick leave, or business leave; (2) nursing managers.

### 2.3 Sample size

To enhance the reliability of the latent profile analysis, a sample size of at least 500 is advocated in research practice. Our study, with a sample size of 832, meets and exceeds this recommendation, thereby strengthening the confidence in our analytic results (Nylund et al., 2007).

### 2.4 Measures

#### 2.4.1 Social–demographic characteristics questionnaire

The questionnaire was designed by the researcher, and the data collected were objective, including demographic characteristics, satisfaction with scheduling and innovation-related information. Demographic characteristics: age, marital status, working years, education level, professional title, average monthly income, satisfaction with scheduling; innovation-related information: experience of innovation, participation in scientific research training, participation in academic conferences, participation in teaching innovation competition, and publication of academic articles.

#### 2.4.2 Paradoxical leadership scale

The scale was developed by Zhang et al. (2015) to measure the status of paradoxical leader behavior in people management and has been widely used among nurses (Salama et al., 2025). The scale consists of five dimensions: maintaining both distance and closeness, maintaining decision control while allowing autonomy, treating subordinates uniformly while allowing individualization, enforcing work requirements while allowing flexibility, and combining self-centeredness with other-centeredness. These 5 dimensions together formed 22 items. Higher scores on the 5-point Likert scale indicated the more representative paradoxical leader behavior. Cronbach’s alpha coefficient for the total scale in this study was calculated as 0.898, indicating strong internal consistency.

#### 2.4.3 Creative self-efficacy scale

The scale was developed by Carmeli (2007) to measure the Creative Self-efficacy of respondents. (Yuandong and Jisheng, 2010) revised and improved it according to the actual situation in China, arguing that creative self-efficacy refers not only to the belief in obtaining the results of innovation but also should include the belief in taking a creative approach to work. The scale was a single dimension with 8 items, and each item was scored using a 5-point Likert scale, where one represents strongly disagree and five represents strongly agree. The higher the total score, the higher the level of creative self-efficacy. Cronbach’s alpha coefficient for the total scale in this study was calculated as 0.906.

#### 2.4.4 Work engagement scale

The scale was developed by Schaufeli et al. (2006) and translated into Chinese and revised by Fu-ye et al. (2013), which was widely used in Chinese nurses (Zhang et al., 2025). The Work Engagement Scale is used to evaluate the attitude, performance, and the respondents’ satisfaction with their work, reflecting their involvement, dedication, and enthusiasm for their jobs. The Chinese version of the Work Engagement Scale consists of three dimensions: vigor, dedication, and absorption, with three items for each dimension, totaling nine items. Each item is scored on a 7-point Likert scale, and higher scores indicate a higher level of work engagement. In this study, the overall Cronbach’s *α* coefficient for the scale is 0.895.

#### 2.4.5 The nurse innovation behavior scale

The scale was developed by Scott and Bruce (1994) and compiled by Ling et al. (2012) based on the characteristics of Chinese nurses to investigate the innovative behavior of nurses. It included three dimensions: Generating ideas, Obtaining support and Realizing ideas. Higher scores on the 5-point Likert scale indicated higher innovative behavior. The Cronbach’s alpha coefficient for the total scale in this study was calculated as 0.821.

### 2.5 Data collection

The investigators coordinated with the hospital management department, and with the consent of the department head, the questionnaires were distributed to the nurses. Prior to commencing the formal survey, the researchers provided participants with a detailed explanation of the survey’s objectives and content. They were explicitly informed of their right to voluntarily participate or decline to answer any questions. Participants were assured that the survey was confidential, with all data collected being used solely for scientific research. In total, 890 questionnaires were distributed, and 832 valid questionnaires were collected (excluding those with obvious errors, incorrect answers to lie tests, or incomplete responses). The effective response rate was 92.47%.

### 2.6 Ethics statement

Ethical approval for this study was obtained from the School of Public Health and Nursing, Hangzhou Normal University, Hangzhou, China (ethical review NO. 2024039), based on the principles of the Declaration of Helsinki. All eligible nurses were informed of the study and its ethical principles. Participants were fully informed about the study and voluntarily participated. Specifically, to protect the privacy of the participants, this study anonymized them. The questionnaires did not collect any information that could identify individuals. The collected data was stored in password-protected computers and backed up through encrypted storage devices. The data will be retained for 5 years after the final publication of the paper for possible verification. After the retention period expires, the data will be permanently deleted from all storage devices.

### 2.7 Data analysis

The statistical software M-plus 8.3, IBM SPSS 26.0 and R4.5.1 were used for data analysis.

The M-plus 8.3 software was employed in the LPA process. The LPA was used to identify subgroups of individuals who share a similar profile of scores on nurses’ innovative behavior. The mean variables of nurses’ innovative behavior were measured to assess the trait indicator variables. The Akaike Information Criterion (AIC), Bayesian Information Criterion (BIC), adjusted BIC (aBIC), and an Entropy test were used to select the model. The entropy (entropy) is a measure of the accuracy of model classification is an evaluation index of model classification accuracy. The Bootstrap Likelihood Ratio Test (BLRT), Lomond-Dale-Rubin corrected likelihood ratio (LMR) and adjusted LMR (ALMR), were used to compare the difference in fit between the models (Vrieze, 2012). A stepwise approach was used to determine the number of latent profiles that best characterize the data and sample. The best model is characterized by the following criteria: when AIC, BIC, and aBIC in the model are minimized; entropy value is greater than 0.8; the statistical significance of LMR, ALMR, and BLRT is less than 0.05 indicates that the k-category model is better than the k-1-category model; the profiles are both concise and interpretable (Wang and Bi, 2018).

IBM SPSS 26.0 software was employed to conduct statistical analysis. Descriptive statistics were presented as frequency and percentage for categorical data, and mean and standard deviation for continuous data. To identify differences in innovative behavior profiles based on socio-demographic and professional characteristics, Pearson’s *χ*^2^ test was used for categorical variables, and one-way ANOVA was used for continuous variables. Prior to conducting ANOVA, its assumptions were assessed. The normality of the distribution for all continuous variables (including Likert-scale scores and subscales) was evaluated by examining skewness and kurtosis; In this study, the absolute values were, respectively, lower than 2 and 4, indicating that the data conformed to a normal distribution. Homogeneity of variances was tested using Levene’s test. If the normality assumption was violated, the non-parametric Kruskal-Wallis test was applied as an alternative. For comparisons that met the normality assumption but violated homogeneity of variances (Levene’s test *p* < 0.05), Welch’s ANOVA was reported.

In order to deeply explore the key predictors affecting different innovation behavior profiles, this study used Random Forest machine learning algorithm with R4.5.1 and R Studio to rank the factors influencing innovative behaviors of different subgroups (C1, C2, and C3) in terms of importance respectively, and assessed their innovative performance and feature importance.

1. *Data preprocessing and sampling strategy*: The analysis was based on the three populations that were classified by the latent profile analysis described above. For each profile, we first constructed a dichotomous dataset (e.g., for the “C1” subgroup, labeled as “C1” = 1 and “non-C1” = 0). To effectively address the category imbalance problem, a stratified sampling strategy is used in the model training phase to ensure that the sample sizes of the target and non-target categories in the training set are equal, which can optimize the model’s ability to recognize a small number of categories. The dataset was randomly divided into training set and test set in the ratio of 7:3.
2. *Model training and feature significance assessment*: random forest models were constructed using the rfPermute package in the R4.5.1. The package adds a significance assessment function based on the permutation test to the standard random forest algorithm, which is able to provide accurate *p*-values for the computed feature significance and greatly enhances the statistical robustness of the results. Each model is set up with 500 ntree and 1,000 permutation repetitions for each feature variable to reliably estimate its significance. Feature importance is measured by the average Gini reduction, which represents the average value of a feature’s contribution to node purity in the model, with larger values indicating that the feature is more important for predictive classification.
3. *Model performance was evaluated using accuracy, sensitivity, and AUC*: Accuracy assessed the overall correct classification rate, while sensitivity measured the model’s ability to correctly identify nurses belonging to the target latent profile of innovative behavior. The calculation of AUC is based on the subject operating characteristic curve (ROC curve), which is a metric used to evaluate the performance of binary classification models, and is often used to evaluate the classification performance of unbalanced samples.
4. *Visualization*: For each profile, the significance of the feature variables was extracted and bar charts with significance markers (*p < 0.05, **p < 0.01, ***p < 0.001) ranking the significance of the features were plotted to visualize the relative contribution of each predictor. A two-sided *p*-value < 0.05 was considered statistically significant.

### 2.8 Multicollinearity and common method bias

Since there are many variable questionnaires involved in this study, all filled in by the same respondent, there may be common method variance. This study was tested by the Harman univariate test. The results showed that the percentage of variance explained by the first common factor was 31.016%, less than 40%, and there were 11 common factor eigenvalues greater than 1, so there was no serious common method bias problem (Tang and Wen, 2020).

## 3 Results

### 3.1 General information on respondents

Descriptive and univariate analyses of innovation behavior among nurses were performed based on data collected from 832 participants. The majority of participants in this study were women (*n* = 794, 95.43%), predominantly aged between 31 and 40 years (*n* = 401, 48.20%). Other general information is shown in Table 1.

**Table 1:** The differences in nurses’ innovative behavior in social–demographic characteristics, paradoxical leadership, creative self-efficacy, and work engagement.

Variables		Overall	P1: Innovation-Conservative (13.34%)	P2: Innovation-Exploratory (65.50%)	P3: Innovation-Driven (21.16%)	*χ*^2^/*F*	*p*
Gender	Male	38	3 (2.70%)	25 (4.59%)	10 (5.68%)	1.387^a^	0.500
	Female	794	108 (97.30%)	520 (95.41%)	166 (94.32%)		
Age	18–30	235	78 (70.27%)	131 (24.04%)	26 (14.77%)	139.395^a^	0.000
	31–40	401	25 (22.52%)	297 (54.50%)	79 (44.89%)		
	41–50	167	7 (6.31%)	101 (18.53%)	59 (33.52%)		
	>50	29	1 (0.90%)	16 (2.93%)	12 (6.82%)		
Marital status	Unmarried	176	21 (18.92%)	117 (21.47%)	38 (21.59%)	10.125^a^	0.038
	Married	639	84 (75.68%)	417 (76.51%)	138 (78.41%)		
	Divorced and widowed	17	6 (5.40%)	11 (2.02%)	0 (0.00%)		
Number of children	0	238	33 (29.73%)	158 (28.99%)	47 (26.70%)	5.002	0.516
	1	360	42 (37.84%)	235 (43.12%)	83 (47.16%)		
	2	227	34 (30.63%)	149 (27.34%)	44 (25.00%)		
	≥3	7	2 (1.80%)	3 (0.55%)	2 (1.14%)		
Working years	<5	111	52 (46.85%)	52 (9.54%)	7 (3.98%)	188.487^a^	0.000
	5–10	255	47 (42.34%)	166 (30.46%)	42 (23.86%)		
	11–20	332	9 (8.11%)	250 (45.87%)	73 (41.48%)		
	>20	134	3 (2.70%)	77 (14.13%)	54 (30.68%)		
Education level	Associate degree and below	63	38 (34.23%)	23 (4.22%)	2 (1.14%)	148.794^a^	0.000
	Bachelor’s degree	738	73 (65.77%)	507 (93.03%)	158 (89.77%)		
	Master’s degree and above	31	0 (0.00%)	15 (2.75%)	16 (9.09%)		
Department	Internal medicine	208	35 (31.53%)	134 (24.59%)	39 (22.16%)	11.874^a^	0.456
	Surgery	230	25 (22.52%)	151 (27.71%)	54 (30.68%)		
	Obstetrics and gynecology	66	3 (2.70%)	47 (8.62%)	16 (9.09%)		
	Pediatrics	46	6 (5.41%)	29 (5.32%)	11 (6.25%)		
	ICU and emergency	116	15 (13.51%)	79 (14.50%)	22 (12.50%)		
	Operating room	57	7 (6.31%)	39 (7.16%)	11 (6.25%)		
	Others	109	20 (18.02%)	66 (12.10%)	23 (13.07%)		
Employment type	Personnel on payroll	621	80 (72.07%)	405 (74.31%)	136 (77.27%)	1.062^a^	0.588
	Others	211	31 (27.93%)	140 (25.69%)	40 (22.73%)		
Professional title	Primary nurse	61	36 (32.43%)	24 (4.40%)	1 (0.57%)	173.255^a^	0.000
	Senior nurse	421	67 (60.36%)	286 (52.48%)	68 (38.64%)		
	Supervisor nurse	294	7 (6.31%)	204 (37.43%)	83 (47.16%)		
	Co-chief nurse and above	56	1 (0.90%)	31 (5.69%)	24 (13.63%)		
Night shift per month	0	113	17 (15.32%)	74 (13.58%)	22 (12.50%)	0.830^a^	0.934
	1–5	327	40 (36.04%)	216 (39.63%)	71 (40.34%)		
	>5	392	54 (48.64%)	255 (46.79%)	83 (47.16%)		
Average monthly income (Chinese yuan)	<4,000	35	16 (14.41%)	13 (2.39%)	6 (3.41%)	86.359^a^	0.000
	4,000–6,000	200	47 (42.34%)	122 (22.39%)	31 (17.61%)		
	6,001–8,000	312	32 (28.83%)	217 (39.82%)	63 (35.80%)		
	8,001–10,000	230	13 (11.71%)	166 (30.46%)	51 (28.98%)		
	>10,000	55	3 (2.71%)	27 (4.94%)	25 (14.20%)		
Satisfaction with scheduling	Very unsatisfied	10	3 (2.70%)	2 (0.37%)	5 (2.84%)	53.886^a^	0.000
	Quite unsatisfied	22	11 (9.91%)	8 (1.47%)	3 (1.70%)		
	Generally satisfied	197	38 (34.23%)	130 (23.85%)	29 (16.48%)		
	Quite satisfied	356	40 (36.04%)	241 (44.22%)	75 (42.61%)		
	Very satisfied	247	19 (17.12%)	164 (30.09%)	64 (36.37%)		
Experience of innovation	Yes	428	41 (36.94%)	285 (52.29%)	102 (57.95%)	12.496^a^	0.002
	No	404	70 (63.06%)	260 (47.71%)	74 (42.05%)		
Participation in scientific research training	Yes	299	28 (25.23%)	195 (35.78%)	76 (43.18%)	9.551^a^	0.008
	No	533	83 (74.77%)	350 (64.22%)	100 (56.82%)		
Participation in academic conferences	Yes	290	24 (21.62%)	201 (36.88%)	65 (36.93%)	9.880^a^	0.007
	No	542	87 (78.38%)	344 (63.12%)	111 (63.07%)		
Participation in teaching innovation competition	Yes	47	1 (0.90%)	29 (5.32%)	17 (9.66%)	10.115^a^	0.006
	No	785	110 (99.10%)	516 (94.68%)	159 (90.34%)		
Initiation of the project	Yes	74	9 (8.11%)	43 (7.89%)	22 (12.50%)	3.587^a^	0.166
	No	758	102 (91.89%)	502 (92.11%)	154 (87.50%)		
Publication of academic articles	Yes	171	15 (13.51%)	106 (19.45%)	50 (28.41%)	10.427^a^	0.005
	No	661	96 (86.49%)	439 (80.55%)	126 (71.59%)		
Approval of the patent	Yes	60	6 (5.41%)	37 (6.79%)	17 (9.66%)	2.262^a^	0.323
	No	772	105 (94.59%)	508 (93.21%)	159 (90.34%)		
Paradoxical leadership	-	63.28 ± 10.91	46.66 ± 14.01	64.99 ± 7.92	68.48 ± 5.94	123.443^c^	0.000
Maintaining both distance and closeness	-	11.70 ± 2.92	8.57 ± 3.95	12.13 ± 2.35	12.33 ± 2.48	44.122^c^	0.000
Maintaining decision control while allowing autonomy	-	11.44 ± 2.93	8.78 ± 3.78	11.68 ± 2.59	12.35 ± 2.33	39.761^c^	0.000
Treating subordinates uniformly while allowing individualization	-	14.51 ± 3.56	10.59 ± 4.50	14.90 ± 2.86	15.76 ± 3.21	56.747^c^	0.000
Enforcing work requirements while allowing flexibility	-	11.20 ± 3.00	8.33 ± 3.89	11.42 ± 2.56	12.32 ± 2.49	46.301^c^	0.000
Combining self-centeredness with other-centeredness	-	14.45 ± 3.39	10.39 ± 4.67	14.86 ± 2.69	15.72 ± 2.36	62.396^c^	0.000
Creative self-efficacy	-	28.84 ± 5.35	20.83 ± 4.97	29.17 ± 4.10	32.87 ± 3.26	263.537^c^	0.000
Work engagement	-	30.99 ± 8.00	20.16 ± 6.64	31.51 ± 6.61	36.24 ± 6.09	212.502^b^	0.000
Vigor	-	10.05 ± 3.15	6.31 ± 2.86	10.28 ± 2.69	11.72 ± 2.77	139.286^b^	0.000
Dedication	-	10.73 ± 3.14	7.33 ± 3.08	10.83 ± 2.79	12.53 ± 2.48	121.320^b^	0.000
Absorption	-	10.21 ± 3.22	6.52 ± 3.06	10.39 ± 2.86	11.99 ± 2.47	125.032^c^	0.000

^a^ Chi-square test; ^b^ homoscedasticity; one-way ANOVA; ^c^ heteroscedasticity, Welch’s ANOVA.

### 3.2 Identification of nurses’ innovative behavior profiles

The score of each entry of 832 nurses’ innovative behavior was (3.58 ± 0.48) and the scores of the entries of the three dimensions Generating Ideas, Realizing Ideas, and Obtaining Support were 3.74 ± 0.63, 3.54 ± 0.68, and 3.49 ± 0.63, respectively. In this study, 10 entry scores of nurses’ innovative behavior were used as exogenous indexes, and 1 to 5 models were fitted sequentially, and the results of the fitting indices of each model are shown in Table 2. The AIC, BIC and aBIC indices decreased with the increase of the number of categories, the entropy of categories 2 to 5 were all >0.800, the LMR and LMRT indices of categories 2 and 3 were significant, and when the number of categories was 4, LMR(*P*) was not significant. The entropy was 0.841 when the number of categories was 3, and the LMR and BLRT indices reached a significant level (*p* < 0.001). After comprehensively comparing the fitting indexes of each model, Model 3-profile was selected as the best fitting model. We analyzed the posterior probability for the 3 categories, reaching 0.955, 0.929, and 0.915, respectively, which indicated the results of the optimal model obtained are reliable and have a high discriminative ability.

**Table 2:** Model fit comparison of latent profile analysis.

Model	AIC	BIC	aBIC	Entropy	LMR (*p*)	ALMR (*p*)	BLRT (*p*)	Latent profile proportions (%)
1-profile	18905.36	18999.837	19452.303	—	—	—	—	—
2-profile	16884.297	17030.736	16932.291	0.937	0.0000	0.0000	0.0000	16.59/83.41
3-profile	**16156.431**	**16354.832**	**16221.454**	**0.841**	**0.0003**	**0.0003**	**0.0000**	**13.34/65.50/21.16**
4-profile	15892.149	16142.512	15974.202	0.809	0.0559	0.0576	0.0000	10.82/35.7/34.86/18.63
5-profile	15663.313	15965.638	15762.396	0.809	0.2761	0.2796	0.0000	7.81/6.61/24.16/36.18/25.24

aBIC, adjusted Bayesian information criterion; AIC, Akaike information criterion; BIC, Bayesian information criterion; BLRT, the bootstrap likelihood ratio test; LMR, the Lo–Mendell–Rubin test. Model 3-profile was selected as the best fitting model after comprehensively comparing the fitting indexes of each model.

### 3.3 Characterization of potential profiles of nurses’ innovative behavior

**Figure 1:**
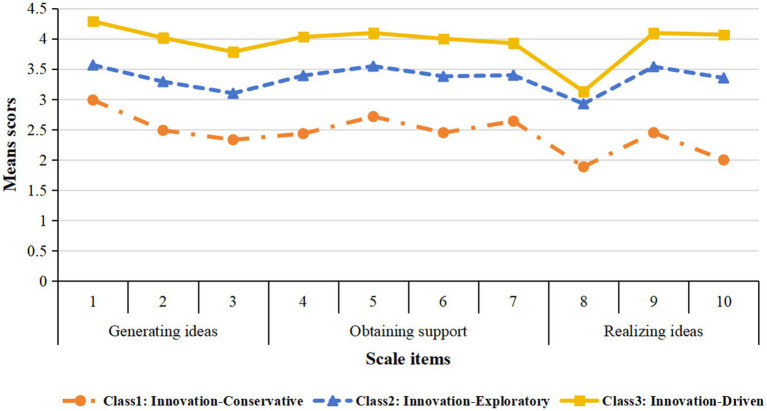
Potential profile of nurses’ innovative behavior.

Based on the classification results, the latent profiles of nurses’ innovative behavior were plotted in Figure 1. The horizontal axis represents the 10 items of nurse innovative behavior and the vertical axis represents their corresponding scores. The higher the item score, the better the level of innovative behavior of nurses. Meanwhile, the profiles were named by combining the characteristics of the item scores. From the results in Figure 1, it can be seen that there are 111 nurses in profile C1 (13.34%). The lowest scores on all dimensions indicate that this subgroup tends to be cautious and traditional in its innovative behavior, with an initial awareness of the problem, but a weak willingness and ability to explore and analyze solutions on their own initiative, and the least ability to turn ideas into reality. Hence the name “Innovation-Conservative”; There are 545 nurses in profile C2 (65.50%). This subgroup scores moderately well on all dimensions. It is a stable, reliable group that lacks prominence and is the backbone of the nurse community that is actively experimenting and exploring new approaches. Hence the name “Innovation-Exploratory”. There are 176 nurses in profile C3 (21.16%). This subgroup is much higher than the other two groups on all dimensions. They are a source of new ideas and are extremely good at promoting their ideas, obtaining support and developing solutions, but even so, finalizing the idea is the most challenging part of the innovation process. As such, this subgroup is a key driver and leader of innovation. Hence the name “Innovation-Driven.”

### 3.4 Demographics and characteristics by latent profile

Table 1 lists the socio-demographic and professional characteristics associated with latent profiles of innovative behavior. The results of univariate analysis showed that there were significantly different in the three latent profiles: age (*p* < 0.001), marital status (*p* < 0.05), working years (*p* < 0.001), education level (*p* < 0.001), professional title (*p* < 0.001), average monthly income (*p* < 0.001), satisfaction with scheduling (*p* < 0.001), experience of innovation (*p* < 0.01), participation in scientific research training (*p* < 0.01), participation in academic conferences (*p* < 0.01), participation in teaching innovation competition (*p* < 0.01), publication of academic articles (*p* < 0.01), paradoxical leadership (*p* < 0.001), creative self-efficacy (*p* < 0.001), and work engagement (*p* < 0.001). The scores of all dimensions of nurses’ innovative behavior, paradoxical leadership, creative self-efficacy, and work engagement in the “Innovation-Conservative” were significantly lower than the overall level. Nurses in the “Innovation-Conservative” accounted for a larger proportion in terms of age 18–30, working years <5 years, relatively low professional title, lower monthly income, and lack of innovative experiences. The proportion of nurses in the “Innovation-Exploratory” was the largest and the nurses in this subgroup were mainly composed of 31–40 year-olds, quite satisfied with nurse scheduling, and had worked for 11–20 years. The score of each dimension in the “Innovation-Driven” was the highest. In this subgroup, the composition of the nurses was dominated by those with the title of supervisor nurse or above, more than 20 years of working, higher income levels, and richer experience in innovation. Furthermore, compared to the “Innovation-Conservative” and “Innovation-Exploratory” subgroups, the “Innovation-Driven” had significantly higher scores in paradoxical leadership, creative self-efficacy, and work engagement.

### 3.5 Importance ranking of factors influencing nurses’ innovative behavior based on random forest model

For the random forest model performance test, we measured the accuracy value of 0.90, 0.71, and 0.75, sensitivity value of 0.85, 0.75, and 0.75, and AUC of 0.94, 0.76, and 0.82 for the three latent classes of Innovation-Conservative, Innovation-Exploratory, and Innovation-Driven. The results of these metrics are acceptable (Huang and Ling, 2005).

**Figure 2:**
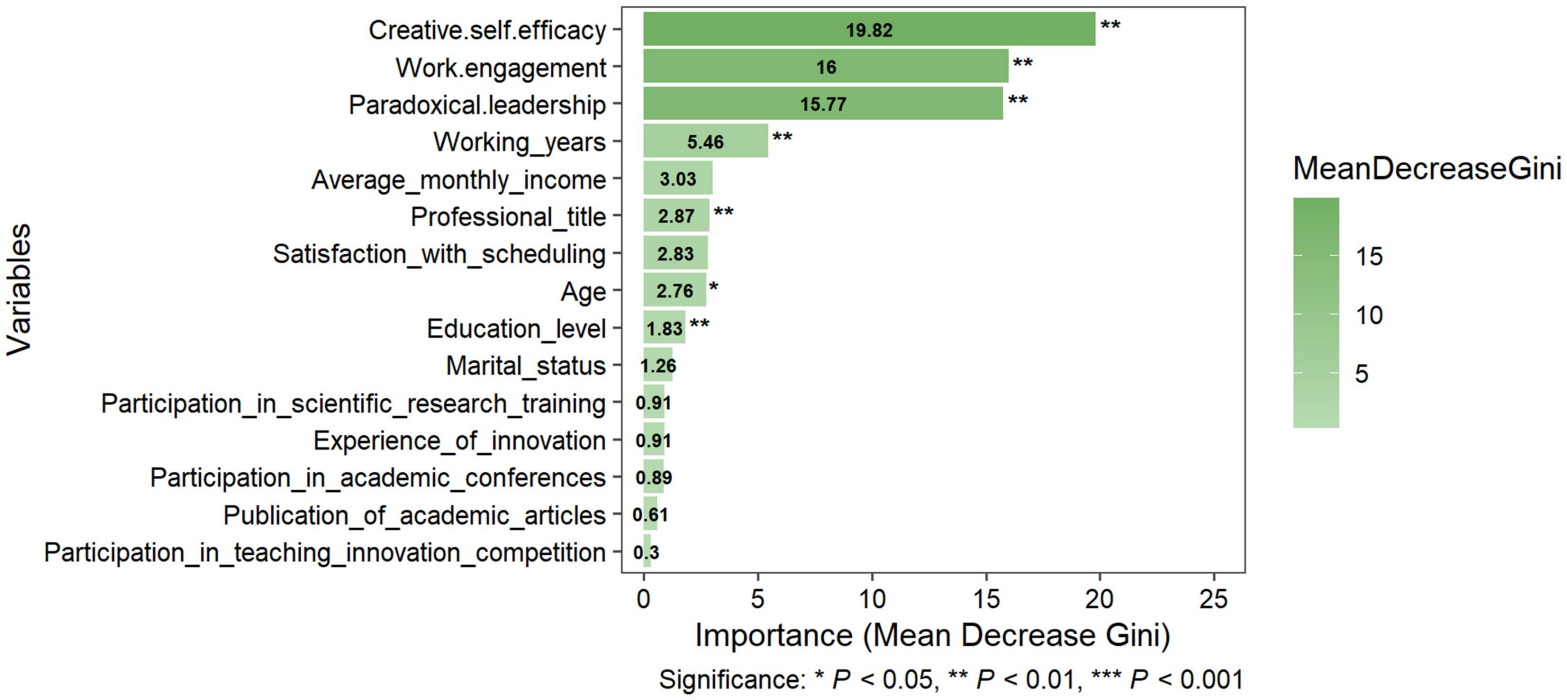
Variable importance for Innovation-Conservative.

**Figure 3:**
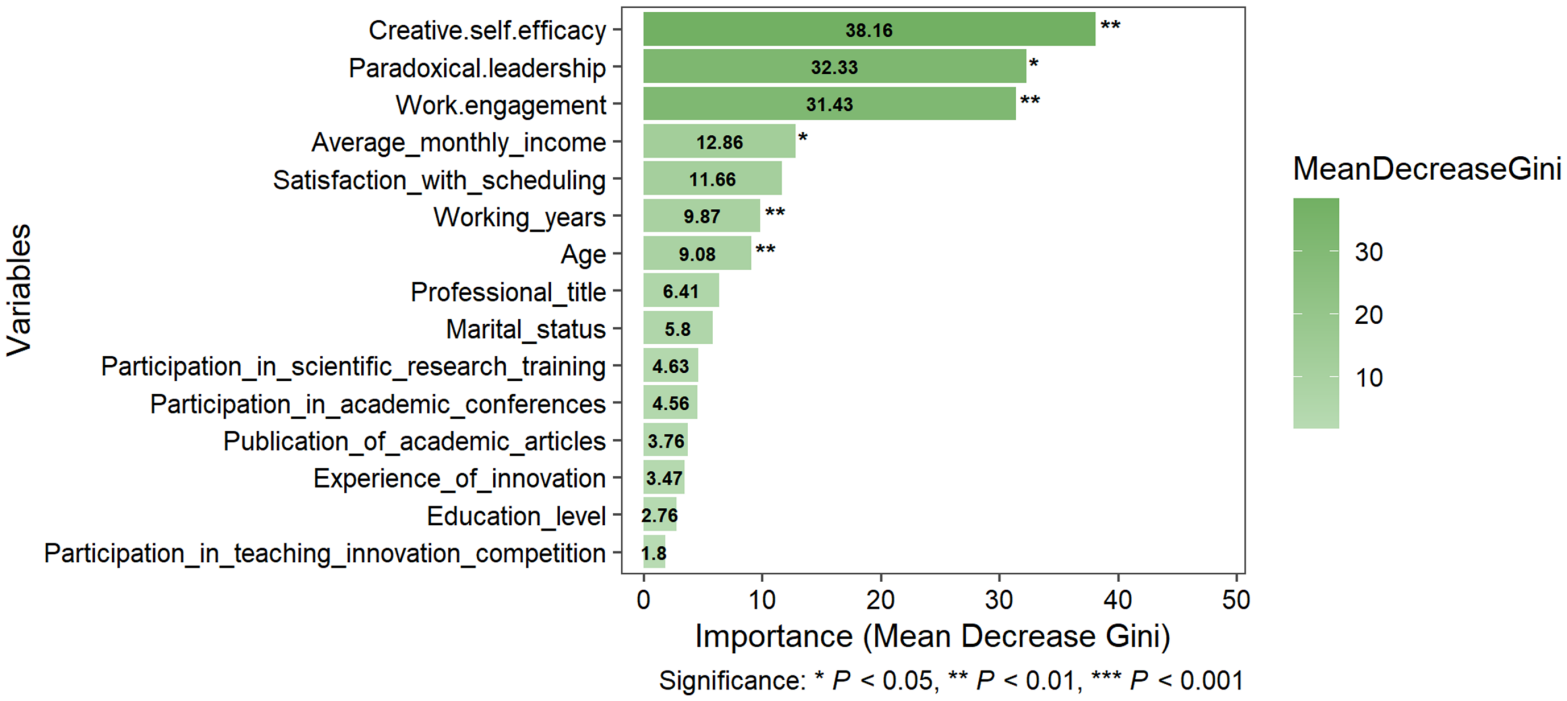
Variable importance for Innovation-Exploratory.

**Figure 4:**
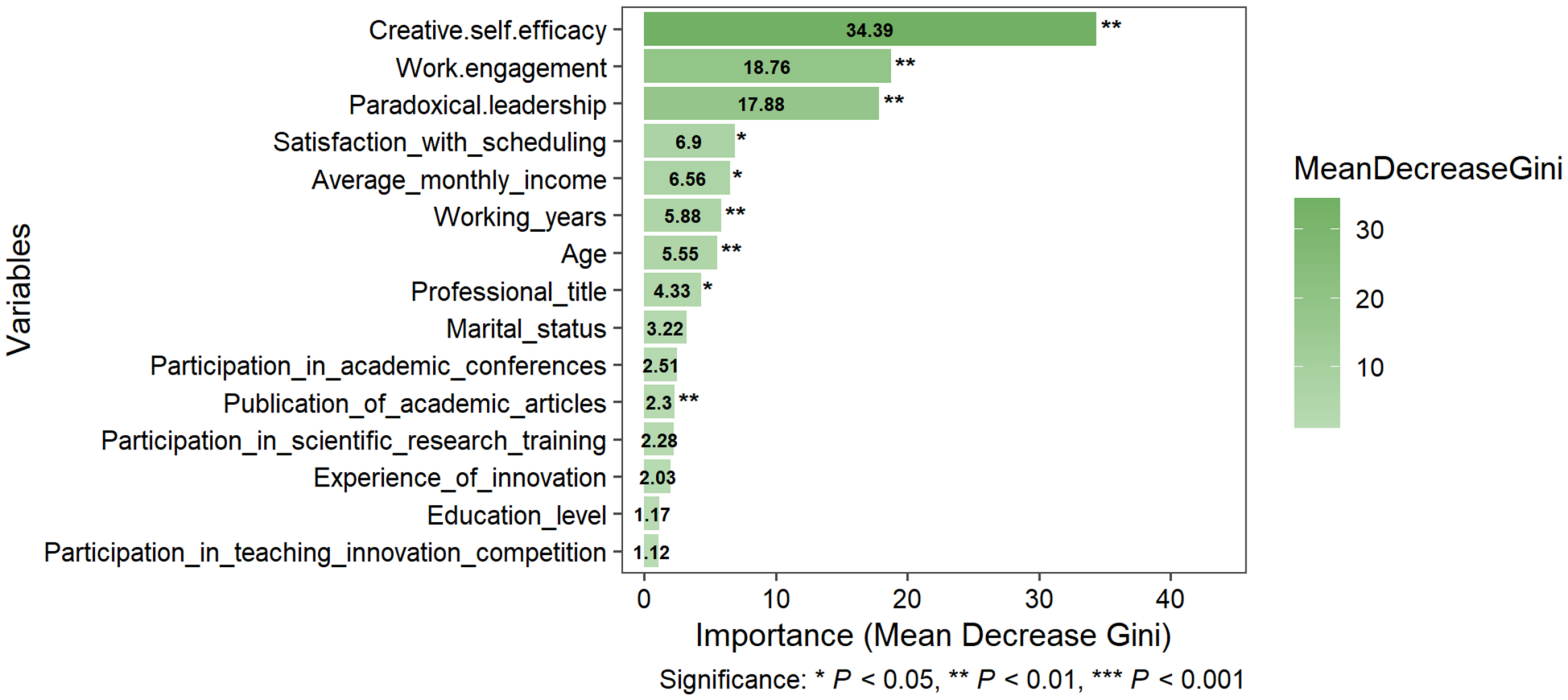
Variable importance for Innovation-Driven.

After training the algorithm, the variables’ feature importance scores were plotted for “Innovation-Conservative,” “Innovation-Exploratory,” and “Innovation-Driven.” The feature importance ranking is demonstrated in Figures 2–4, respectively. The y-axis represents the explanatory variables, while the x-axis represents the feature importance score. A total of 15 variables were ranked from highest to lowest.

Despite differences in specific rankings, creative self-efficacy, work engagement, and paradoxical leadership were consistently identified as the top three most critical features across all subgroups, with differences in subsequent rankings: the variables of working years, average monthly income, and satisfaction with scheduling, while all ranking in the top five of all subgroups, differed in their specific importance rankings.

For the “Innovation-Conservative,” the five most important predictors are listed in descending order: creative self-efficacy (Mean Decrease Gini: 19.82, *p* < 0.01), work engagement (Mean Decrease Gini: 16.00, *p* < 0.01), paradoxical leadership (Mean Decrease Gini: 15.77, *p* < 0.01), working years (Mean Decrease Gini: 5.46, *p* < 0.01), and average monthly income (Mean Decrease Gini: 3.03, *P* >0.05). Other significant predictors included professional title (Mean Decrease Gini: 2.87, *p* < 0.01), age (Mean Decrease Gini: 2.76, *p* < 0.05), and education level (Mean Decrease Gini: 1.83, *p* < 0.01), seeing Figure 2.

For the “Innovation-Exploratory,” the five most important predictors were: creative self-efficacy (Mean Decrease Gini: 38.16, *p* < 0.01), paradoxical leadership (Mean Decrease Gini: 32.33, *p* < 0.05), work engagement (Mean Decrease Gini: 31.43, *p* < 0.01), average monthly income (Mean Decrease Gini: 12.86, *p* < 0.05), and satisfaction with scheduling (Mean Decrease Gini: 11.66, *p* > 0.05). The predictors working years (Mean Decrease Gini: 9.87, *p* < 0.01) and age (Mean Decrease Gini: 9.08, *p* < 0.01) were also statistically significant, seeing Figure 3.

For the “Innovation-Driven,” the five most important predictors were: creative self-efficacy (Mean Decrease Gini: 34.39, *p* < 0.01), work engagement (Mean Decrease Gini: 18.76, *p* < 0.01), paradoxical leadership (Mean Decrease Gini: 17.88, *p* < 0.01), satisfaction with scheduling (Mean Decrease Gini: 6.90, *p* < 0.05), and average monthly income (Mean Decrease Gini: 6.56, *p* < 0.05). Significant predictors also included working years (Mean Decrease Gini: 5.88, *p* < 0.01), age (Mean Decrease Gini: 5.55, *p* < 0.01), professional title (Mean Decrease Gini: 4.33, *p* < 0.05), and publication of academic articles (Mean Decrease Gini: 2.30, *p* < 0.05), seeing Figure 4.

## 4 Discussion

### 4.1 Comparison of innovative behavior scores and profile distribution

The results of this survey showed that the score of the innovative behavior scale for nurses was (35.79 ± 4.84), which is slightly above the average of previous studies (Wang et al., 2024; Lu et al., 2025; Han and Ma, 2023). Moreover, regarding the latent profile analysis, our findings revealed a distinct distribution of nurses across the three profiles Innovation-Conservative (13.34%), Innovation-Exploratory (65.50%), and Innovation-Driven (21.16%). The proportion of nurses in the Innovation-Conservative subgroup was relatively small, while that in the Innovation-Exploratory subgroup was relatively large. This distribution differs notably from the patterns identified in other studies employing LPA (Li et al., 2025; Fu et al., 2024). The differences in these subgroup distributions may be attributed to variations in hospital levels, and regional socio-economic backgrounds. Compared with previous studies, our research was conducted in public tertiary hospitals in Zhejiang Province, which is a region renowned for its economic development and advanced medical system. Meanwhile, tertiary hospitals are equipped with advanced facilities, advocate an open and inclusive working atmosphere that encourages exploration, and attach great importance to scientific research activities and teaching quality, providing a favorable environment for nurses’ innovative behavior.

### 4.2 Factors influencing nurses’ innovative behavior

Creative self-efficacy is the most significant factor showing a positive association with nurses’ innovative behaviors across all subgroups. Theoretically, it is positively related to intrinsic motivation and willingness to engage in innovative behavior. It is also a critical psychological resource for individuals to realize creative work and carries important practical implications for guiding individual creative work. Studies indicate that nurses with high creative self-efficacy are more likely to actively explore new methods, put forward improvement suggestions, and try innovative strategies to solve problems (Yang et al., 2024). From the perspective of COR theory, innovative behavior does not occur naturally. Essentially, it requires nurses to invest their precious personal resources (e.g., time, energy, and cognitive efforts) while also facing uncertain returns and potential failure risks. Therefore, nurses’ decisions to engage in innovation are linked to their assessment of personal resource status. As a key personal resource, creative self-efficacy represents an important component of nurses’ personal resource pool. Nurses with higher creative self-efficacy may possess greater confidence to consider innovative, high-risk investments, as they are more likely to believe in a higher probability of success, potentially lowering their perceived investment risk. Thus, when nurses have a high level of creative self-efficacy and believe they possess the knowledge and skills to support creativity, they may be more autonomous in accepting work challenges and engaging in creative work practices.

Paradoxical leadership employs balanced thinking within chaotic contexts, flexibly adapting styles to achieve “both-and” outcomes. The personalized care and support characteristics of paradoxical leadership align with the needs of contemporary nurses (Qing et al., 2023). Li and Wang (2021) hold that paradoxical leadership is associated with reduced internal team conflict and carries positive implications for team innovative capacity. Leaders with paradoxical leadership could assist nurses in find a balance among multiple contradictions, such as standardized processes and personalized care, efficiency and safety, strict management, and humanistic care. Specifically, this leadership approach may be consistent with addressing individual nurse needs while maintaining managerial efficiency, supporting creativity within standardized workflows, and facilitating evidence-based innovation initiatives from a practical standpoint. Moreover, paradoxical leadership helps nurses better cope with the complexity and uncertainty in their work, reduces role conflicts and stress, and creates an environment that embraces contradictions, encourages attempts, and allows for failure, which is more likely to lower the risk of potential losses from innovative investments. Under the paradoxical leadership style, nurses might perceive that unsuccessful innovative attempts would not lead to the loss of key resources such as leader recognition or professional reputation, which may in turn be associated with reduced hesitation to engage in innovative behaviors.

Work engagement represents a positive work state distinct from job burnout, characterized by sustained energy and passion toward work (Bakker et al., 2008). Work engagement is negatively associated with negative emotions and passive work behaviors commonly observed among nurses experiencing job burnout and work pressure (Lv et al., 2022). Innovative behavior is a complex behavior, which is a series of attempts with uncertainties and risks, requiring knowledge, motivation, and substantial resource investment. Meanwhile, compared with ordinary nurses, those with such a positive state tend to devote more time and energy to their work and show more tolerance for failure and setbacks. Highly engaged nurses demonstrate greater persistence through challenges, actively seek solutions, and invest discretionary effort. These characteristics of nurses are positively associated with the likelihood of engaging in innovative behaviors among nurses (Janssen, 2000). Furthermore, when nurses possess both abundant internal resources and reliable external safeguards, that is, high creative self-efficacy and paradoxical leadership, they tend to enter a state of abundant resources and a sense of security, which is manifested as a high level of organizational commitment (Ling et al., 2012). In such a state, nurses could be more willing to invest their available personal resources to pursue future work-related benefits, including a sense of achievement at work, professional recognition, and career development. This kind of investment tendency align with a greater likelihood of innovative behavior among nursing staff. Conversely, nurses lacking these resources may exhibit a state of resource defense, striving to protect their already scarce resources, and consequently showing a lower tendency to engage in activities that may consume resources, such as innovation. This pattern of associations is consistent with the observation that the Innovation-Conservative subgroup reported the lowest levels of creative self-efficacy, paradoxical leadership, and work engagement.

Beyond these organizational and psychological factors, the random forest feature importance analysis revealed that each subgroup of innovative behavior has its relatively important influencing factors such as working years, average monthly income and satisfaction with nursing work scheduling. Nurses with long working years and high professional title typically possess extensive clinical experience and robust professional knowledge. They care for a large number of patients, hold experience in handling emergencies, and are well familiar with the strengths and weaknesses of various work processes. Innovation is often not about imagination out of thin air, but rather based on the identification and improvement of existing problems. The acute discovery of problems in clinical practice and the generation of innovative ideas are positively linked to rich professional experience. In addition, nurses with long-term clinical practice accumulation are often better able to assess the feasibility of innovative ideas from a practical perspective, which is associated with lower potential risks in the innovation process and makes it more likely for innovative attempts to succeed. Moreover, nurses with rich working experience typically have accumulated a deep professional reputation and interpersonal relationships in their past work. The innovative ideas they propose may be more likely to receive support from colleagues and leaders, which is conducive to the implementation of innovative ideas.

Average monthly income emerged as another factor associated with differential innovative behavior among the nurse profiles. In the Innovation-Conservative subgroup, nurses are usually in the early stage of their careers, characterized by lower professional titles and thus lower incomes. According to the COR theory, nurses with less resource reserves are more prone to the pressure of resource loss. Moreover, the existence of this pressure is linked to the resources running behind their expenses, potentially associated with gradual declines in available resources and gradually increasing the psychological pressure perceived by individuals. Therefore, nurses with lower monthly incomes may have higher survival anxiety and tend to prioritize actions aimed at preventing resource loss, which may create additional barriers to carry out resource investment activities such as innovation. Furthermore, nurses facing financial constraints typically exhibit lower risk tolerance. For these individuals, failure could be associated with additional pressure and negative evaluations from leaders, which may be linked to greater hesitancy toward innovative attempts. Unlike the Innovation-Conservative subgroup, basic livelihood needs among nurses in the Innovation-Exploratory subgroup are generally met, and income serves as an incentive that aligns with organizational recognition and affirmation of their value. This sense of recognition is positively related to stronger organizational commitment and professional identity, which are associated with greater engagement in innovative and other extra-role behaviors as a form of reciprocal contribution to the organization (Joo and Hong, 2026). Ultimately, within the Innovation-Driven subgroup, a competitive income likely acts as a sustaining factor. It helps to bolster their sense of belonging and high engagement, which may be essential for maintaining their long-term innovative output and preventing burnout.

Collectively, our findings point to the potential importance of optimizing the work environment, particularly work scheduling, in fostering nurses’ innovative behavior. Reasonable and efficient work scheduling could help nurses better balance work and life, ensuring adequate rest to maintain the energy required for innovative efforts. Reasonable work scheduling generally involves a certain degree of flexibility and employee participation, which is associated with higher perceived work autonomy among nurses and is positively related to their intrinsic motivation for innovation. For the nurses in the Innovation-Driven subgroup, they scored very high in both the dimensions of generating ideas and obtaining support. However, they encountered obstacles when implementing their ideas. Their scores in item 8 were relatively low, indicating that nurses are likely to have some difficulties in applying the innovative implementation plan to their work. Meanwhile, random forest analysis demonstrates that the satisfaction with work scheduling is a relatively top-ranked factor among the influencing factors of Innovation-Driven subgroup, suggesting that their demand for work scheduling may reflect a sense of job control when implementing innovative behaviors (Joachim Gerich and Weber, 2020). When translating ideas into practice, nurses tend to benefit from greater autonomy in time arrangement, which supports the continuous progression of innovation initiatives. Nurses with a higher sense of control over their work tend to show greater enthusiasm and initiative towards innovative behaviors.

In the random forest analysis, variables reflecting prior innovative experiences such as experience of innovation and publication of academic articles consistently ranked in the lower half of feature importance across all three innovation subgroups. This pattern aligns with COR, which posits that individuals prioritize behaviors supported by immediately available resources. Personal resources like creative self-efficacy, energy resources reflected in work engagement, and conditional resources provided by paradoxical leadership represent immediate, active assets that directly support present innovation efforts. In contrast, past innovative experiences may not constitute presently available resources. Completing a publication or innovation project typically consumes substantial time, cognitive energy, and emotional investment at the time of occurrence. This suggests that the resources associated with past innovative activities may show a weaker correlation with current innovative behavior when not supported by ongoing contextual resources, aligning with their lower predictive importance in our model. Consequently, when proximal resource indicators are included in predictive models, historical experiential variables contribute minimally to explaining current innovation levels. Practically, clinical nursing environments prioritize real time problem solving under time pressure. Furthermore, workload pressures and institutional structures may frequently limit nurses’ opportunities to accumulate publication or competition experiences, making these metrics less representative of frontline innovative capacity. Consequently, experiential indicators demonstrate weaker predictive relevance in this context.

### 4.3 Limitations

This study provides valuable insights into the factors that influence innovative behaviors among nurses. However, in order to contextualize the findings and guide future research, it is important to recognize some limitations. Firstly, this study used a cross-sectional design that involved surveys at a specific point in time. To explore the influencing factors of innovative behaviors of nurses in different subgroups, it is necessary to analyze these variables at different time frames. Future research should use longitudinal studies to empirically analyze the causal relationships between mechanisms. Secondly, the use of a convenience sample from six tertiary hospitals in Zhejiang Province introduces a risk of selection bias, which limits the generalizability of our findings. The results may not be fully representative of nurses working in other regions or in different levels of healthcare institutions. Future studies should employ more diverse and representative sampling strategies across various cultural and healthcare settings to verify the universality of the conclusions.

## 5 Implications

Nursing managers should recognize the critical role of nurses’ innovative behaviors in enhancing healthcare quality and advancing the nursing discipline. This study indicates that providing psychological and environmental support for all nurses is the most important measure to enhance the level of nurses’ innovative behavior. First of all, paradoxical leadership should be cultivated as a competence of nursing managers. By adopting paradoxical leadership approaches while simultaneously improving incentive systems and providing diversified professional development platforms. This integrated strategy will foster sustainable innovation practices in clinical settings. Subsequently, establish a fair performance evaluation and salary system, reward nurses who actively innovate, effectively enhance nurses’ creative self-efficacy and work engagement, and promote the improvement of the overall service performance of the nursing organization and the hospital.

This study emphasizes that working years and average monthly income have both high predictive importance and significant effect on the Innovation-Conservative subgroup, suggesting that professional experience and economic security are the basic conditions for nurses to implement innovative behaviors. Therefore, to address this critical stage, nursing managers should implement strategies to enhance their professional quality and economic security. This includes enhancing clinical skills, providing clear career pathways, ensuring equitable compensation, and giving full play to the role model guidance of the Innovation-Driven subgroup to guide them to complete innovative practices. The Innovation-Exploratory subgroup forming the bulk of the nursing workforce, average monthly income and satisfaction with scheduling emerged as the most important secondary factors. Strategies such as implementing flexible and humanized schedules, reducing administrative burdens, and creating a supportive working environment may be more effective in unleashing their innovative potential than additional training alone. For the Innovation-Driven subgroup, nursing managers should grant full authorization, especially in terms of work scheduling, allowing them to flexibly arrange working time based on the demands of innovative projects. Encourage them to actively disseminate innovative experiences and drive the improvement of the innovation level of the entire nursing team.

## 6 Conclusion

This study employed latent profile analysis and random forest analysis methods to investigate the heterogeneity of innovative behaviors among clinical nurses. Nurses’ innovative behaviors can be classified into three potential profiles: Innovation-Conservative, Innovation-Exploratory, and Innovation-Driven. The results show that the innovative behaviors of nurses are slightly above the average of previous studies. Moreover, utilizing Random Forest algorithm, the research findings suggested that different subgroups were associated with specific combinations of personal and organizational factors. Crucially, the Random Forest algorithm revealed that despite this heterogeneity, adequate creative self-efficacy, work engagement, and paradoxical leadership are the most important factors influencing all subgroups of nurse innovative behavior. Furthermore, working years, average monthly income and satisfaction with scheduling as subordinate factors influence the level of innovative behavior of nurses. Additionally, innovative experience ranks relatively low in the importance ranking.

## Data Availability

The raw data supporting the conclusions of this article will be made available by the authors, without undue reservation.
